# BDSL 49: A comprehensive dataset of Bangla sign language

**DOI:** 10.1016/j.dib.2023.109329

**Published:** 2023-06-18

**Authors:** Ayman Hasib, Jannatul Ferdous Eva, Saqib Sizan Khan, Mst. Nipa Khatun, Ashraful Haque, Nishat Shahrin, Rashik Rahman, Hasan Murad, Md. Rajibul Islam, Molla Rashied Hussein

**Affiliations:** a Department of Computer Science and Engineering, University of Asia Pacific, Dhaka, Bangladesh; b Department of Computer Science and Engineering, Chittagong University of Engineering & Technology, Chittagong, Bangladesh; c Department of Computer Science and Engineering, Bangladesh University of Business and Technology, Dhaka, Bangladesh

**Keywords:** Sign language, Bangla language, Deep learning, Hand configuration, YOLOv4 mode, Computer vision, Natural language processing

## Abstract

Language is a method by which individuals express their thoughts. Each language has its own alphabet and numbers. Oral and written communication are both effective means of human interaction. However, each language has a sign language equivalent. Hearing-impaired and/or nonverbal individuals communicate through sign language. BDSL is the abbreviation for the Bangla sign language. The dataset contains images of hand signs in Bangla. The collection comprises 49 individual sign language images of the Bengali alphabet. BDSL49 is a set of 29,490 images with 49 labels. During data collection, images of fourteen distinct adults, each with a unique appearance and context, were captured. During data preparation, numerous strategies have been utilized to reduce noise. This dataset is available for free to researchers. Using techniques such as machine learning, computer vision, and deep learning, they are able to develop automated systems. Moreover, two models were applied to this dataset. The first is for detection, and the second is for identification.

**Specifications Table**

**Table:** 

Subject	Sign Language, Deep Learning, Computer Vision, Natural Language Processing
Specific subject area	Individuals who have difficulty hearing communicate using sign language. Images of Bangla sign language have been collected to be converted into the Bangla alphabet to improve communication. Techniques such as computer vision, machine learning, deep learning, and natural language processing have been implemented.
Type of data	The detection dataset is in full-frame images. The recognition dataset contains cropped hand sign images, which are in JPG format.
How the data were acquired	Smartphone cameras were used to capture hand gestures corresponding to hand signs.
Data format	Fully labeled and raw RGB images.
Description of data collection	The 49 distinct categories in this dataset are labeled with the Bangla alphabet. Each category contains approximately 600 images, culminating in 29,490 images.
Data source locations	Department of Computer Science and Engineering, University of Asia Pacific, Dhaka, Bangladesh.
Data accessibility	Repository name: BDSL 49: A Comprehensive Dataset of Bangla Sign Language Data identification number: 10.17632/k5yk4j8z8s.6 Direct URL to data: https://data.mendeley.com/datasets/k5yk4j8z8s/6 Instructions for accessing this dataset: This dataset is publicly available for any research, academic, or educational purpose at the repository hyperlink mentioned above.
Related research articles	Begum N, Rahman R, Jahan N, et al. Borno-net: A real-time Bengali sign-character detection and sentence generation system using quantized Yolov4-Tiny and LSTMs. *Appl Sci (Basel)*. 2023;13(9):5219. doi:10.3390/app13095219

## Value of the Data

• The dataset contains a comprehensive set of 29,490 images representing 49 classes of the Bengali alphabet, numeric characters, and special characters. This dataset enables researchers and developers to train and evaluate machine learning models created specifically for identifying Bengali Sign Language hand signs.
• The availability of a Bengali Sign language-specific dataset facilitates research and development in the area of automated sign language recognition. Using this dataset, researchers may create computer-aided models, compare findings, and devise more accurate and robust communication aids for deaf or mute individuals.
• This dataset can be used for training expert systems. The suggested sign language dataset opens up a plethora of new avenues for future study and development. It offers potential advantages in machine learning and artificial intelligence applications.
• The dataset consists of images captured in real-world conditions, reflecting the challenges faced in practical scenarios. This realism helps in training models that can perform well in real-life situations where lighting conditions, backgrounds, and hand positioning may vary.
• This will benefit Bengali speakers as well as those developing translation software to serve as a communication tool for the deaf or the mute.

## 1 Objective

The Bangla hand sign dataset has significant potential as a means of communication for those who are deaf and/or mute. In addition to benefiting the general public and non-disabled individuals who are unfamiliar with sign language, the dataset has the potential to reduce inequity between the deaf or mute populations and non-disabled communities. The main objective of the BDSL 49 dataset is to facilitate increased interaction and accessibility for Bengali Sign Language users. The dataset aims to empower researchers and developers to create automated systems that can accurately recognize and understand the hand signs of Bengali Sign Language. This type of automated system can translate the hand signs of deaf or mute people into text or speech, making it easier for hearing people to comprehend Bengali Sign Language without making any errors. The dataset can also be used for training expert systems, opening up new avenues for study and development and offering advantages in machine learning and artificial intelligence applications. Overall, the Bangla hand sign dataset holds promise for enhancing communication and accessibility for individuals with hearing and speech impairments, as well as advancing technological applications in the field of sign language communication.

## 2 Data Description

About 3 million individuals in Bangladesh are deaf or hearing-impaired [1]. Sign language is a communication technique through which deaf or mute people can communicate with non-disabled people via hand gestures. Verbal communication is quite difficult for deaf and/or mute people. Moreover, they face a significant challenge because non-disabled people fail to understand these complicated hand signs. For this reason, a hand sign dataset based on the Bangla language has been created, which represents the Bangla alphabet and numeric digits. The Bangla Sign Language dataset was created to bridge the gap between deaf and/or mute people and non-disabled communities. This dataset is one of several available in Bangla Sign Language. It helps improve overall accuracy in sign detection and identification. It has been made publicly available to help and encourage more research in Bangla Sign Language Interpretation so that hearing and speech-impaired people can benefit from it. Most of the previous work on the BDSL dataset deals with around 30–38 labels [2], [3], [4], [5], but this dataset deals with 49 labels that have not been applied anywhere else. Two datasets have been produced from these images after acquiring 14,745 images from a few individuals. One is an annotated detection dataset (full-frame image). The second is the recognition dataset (cropped image). This processing of a single image is adapted from [6], and the YOLOv4 methodologies were adapted from [7,8].

**Fig. 1:**
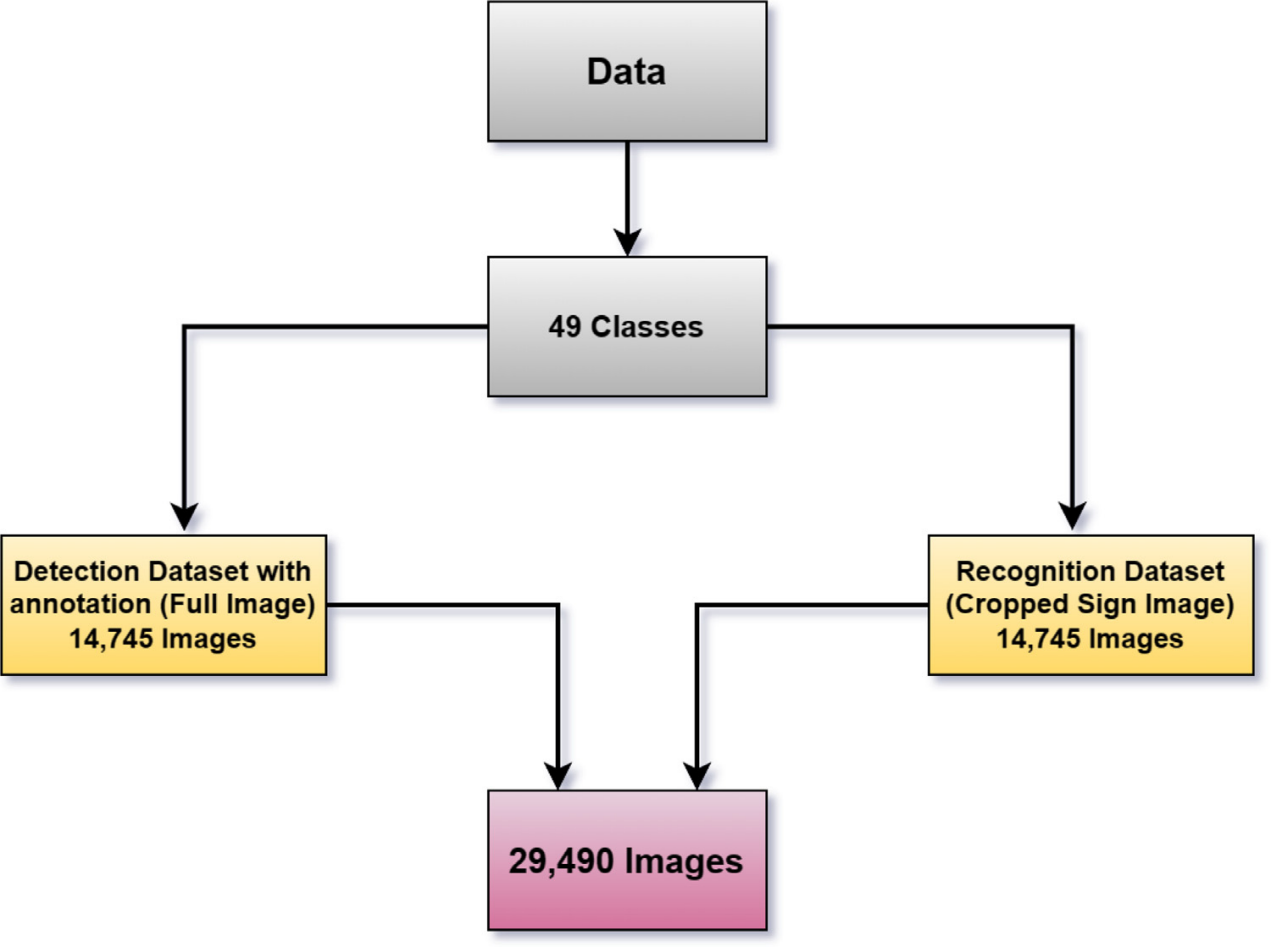
The dataset creation process.

This dataset comprises around 37 alphabets, 10 numeric characters, and 2 special characters to properly recognize Bangla symbols. The dataset consists of 29,490 images divided into two sections, where each section is further divided into 49 classes, as shown in Fig. 1. In this dataset, alphabetic and numeric characters are classified based on the shape and orientation of the hand signals, and samples are attached to the repository link. The first section is for the detection dataset and contains full-frame images, whereas the second section contains solely cropped images of the hand signs for the recognition dataset. In the detection section, each class comprises approximately 300 images captured with various smartphone cameras, for a total of 14,745 images. In the recognition section, images are produced by cropping the hand sign from the full-frame images obtained in the detection section. The entire process is performed with Python and the OpenCV library. Both the detection and recognition datasets are divided into training and testing datasets, with the training dataset including 80% of the total data and the testing dataset containing 20%. The distribution of images among each class is shown in Table 1, where the total number of images is an aggregation of both sections.

**Table 1:** Image distribution among classes considering both sections of the dataset.

**Fig. 2:**
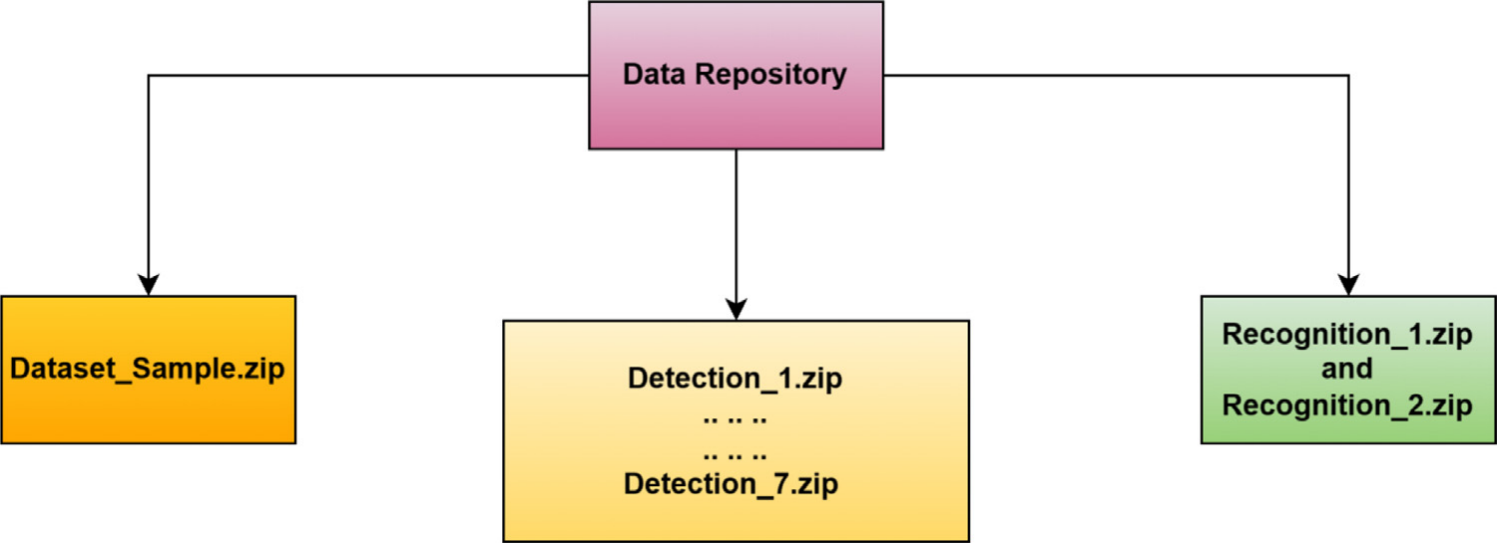
Dataset folders format in data repository.

This study's dataset is accessible through the public repository known as Mendeley Data. There are three different folder categories shown in Fig. 2. The Dataset_Sample.zip folder contains 49 labels, and each level contains a sample image of a hand sign. Detection folders are the second variety. These folders are separated into seven folders titled Detection_1.zip through Detection_7.zip, and each of these seven folders contains seven labels. And collectively, these seven detection folders generate 49 labels for full-frame hand sign images. Recognition folders are the third form of folder, and they are separated into two folders named Recognition_1.zip and Recognition_2.zip. Only cropped images of hand signs are included in these recognition folders. Recognition_1.zip contains almost half of the labels, and Recognition_2.zip contains all the remaining labels. All images in the detection and recognition folders are split into two sets, which are the train folder and the test folder.

## 3 Experimental Design, Materials and Methods

For the creation of this dataset, several participants volunteered to capture images. This dataset contains images captured with various backgrounds. The majority of the images were captured in daylight, but in order to train the model more effectively, some images were captured in dimmer lighting conditions. Different mobile phone cameras with resolutions ranging from 720p to 1080p are utilized for image capture. The camera was set to its default settings, and no additional features were introduced. Automatic exposure was selected for the camera's lighting. By using the above criteria, we captured images for this dataset.

**Fig. 3:**
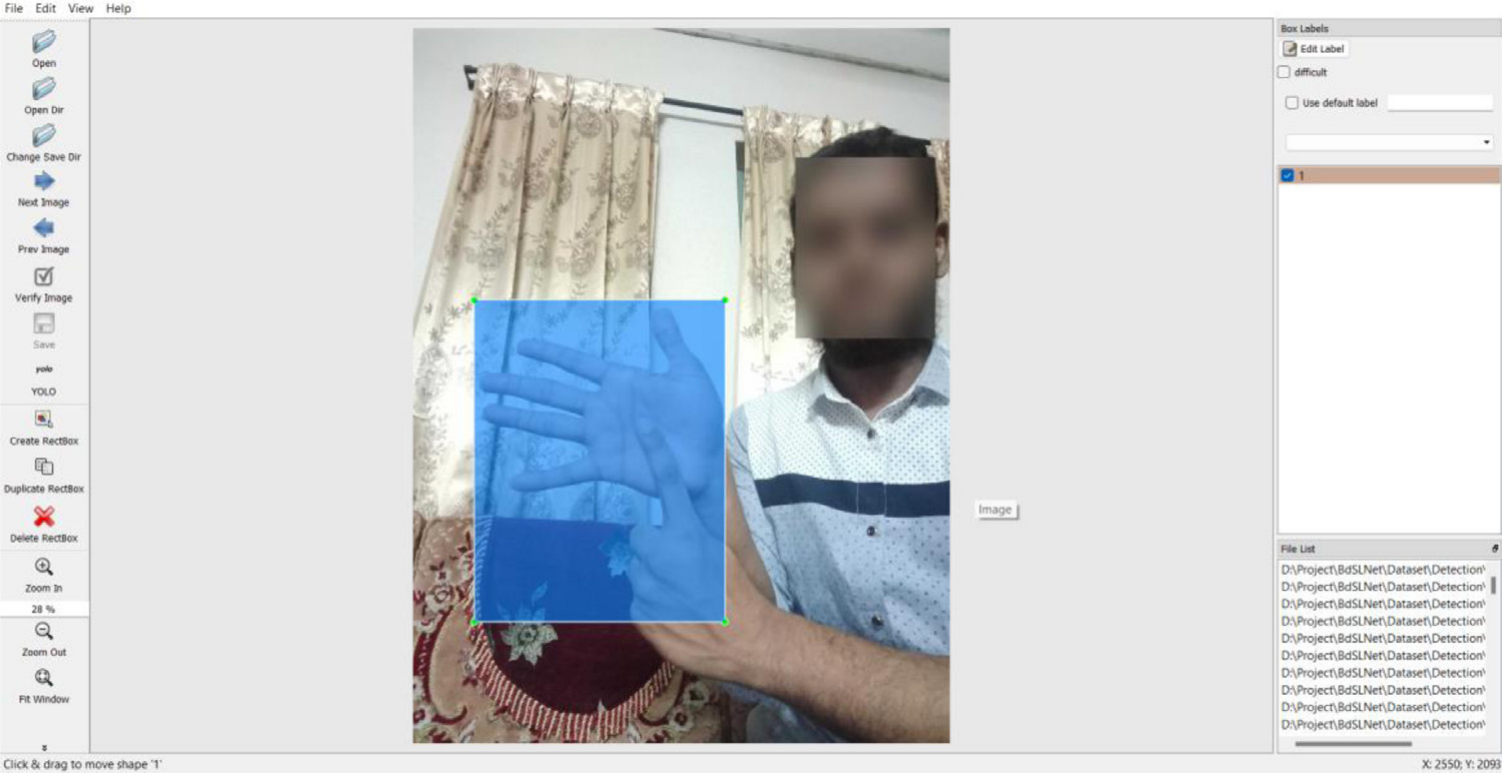
Annotation process.

This dataset has two sections for creating two distinct types of models: one for detection and one for recognition. We utilized full-frame RGB images with varied backgrounds, as shown in Fig. 3, and afterward annotated them to highlight where the sign appeared. As illustrated in this figure, all the faces in the dataset images were detected and blurred using Python and the OpenCV package.

**Fig. 4:**
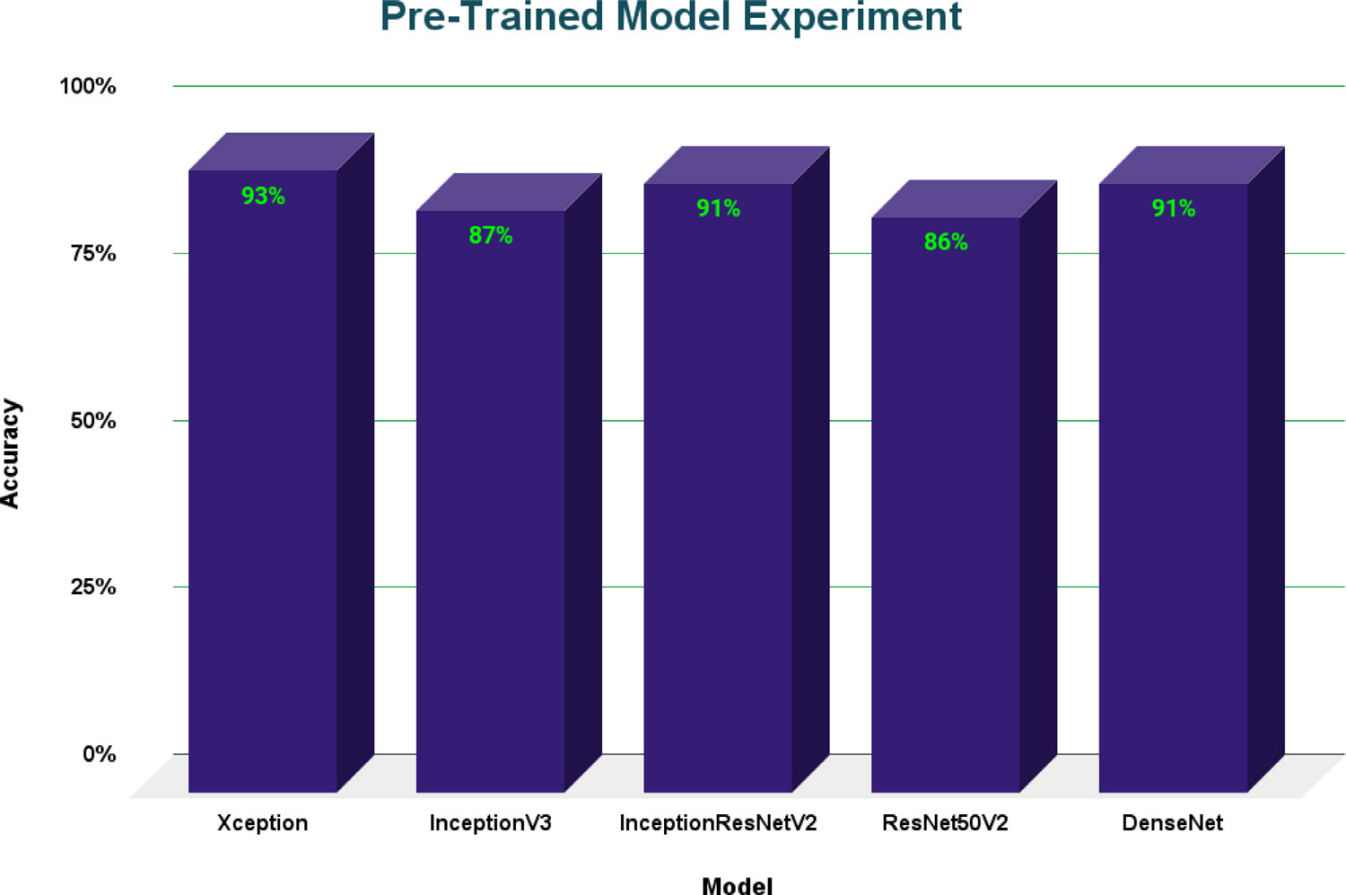
Accuracy comparison of various pre-trained models on the recognition dataset.

Deep learning models were employed in both parts to evaluate model development potential with this dataset. A real-time detection model named YOLOV4 was used for the detection part, and the model attained an accuracy of 99.4%. For recognition, pre-trained models such as Xception, InceptionV3, InceptionResNetv2, ResNet50V2, and DenseNet were employed. Fig. 4 shows accuracy comparisons for all of the models that were trained and assessed on the same dataset.

Here we have implemented various pre-trained models on the recognition dataset and achieved 93% accuracy in Xception, 87% accuracy in InceptionV3, 91% accuracy in InceptionResNetV2, 86% accuracy in ResNet50V2, and 91% accuracy in DenseNet. From this figure, it is to be noted that the Xception model gets the highest accuracy in recognizing the sign languages.

**Fig. 5:**
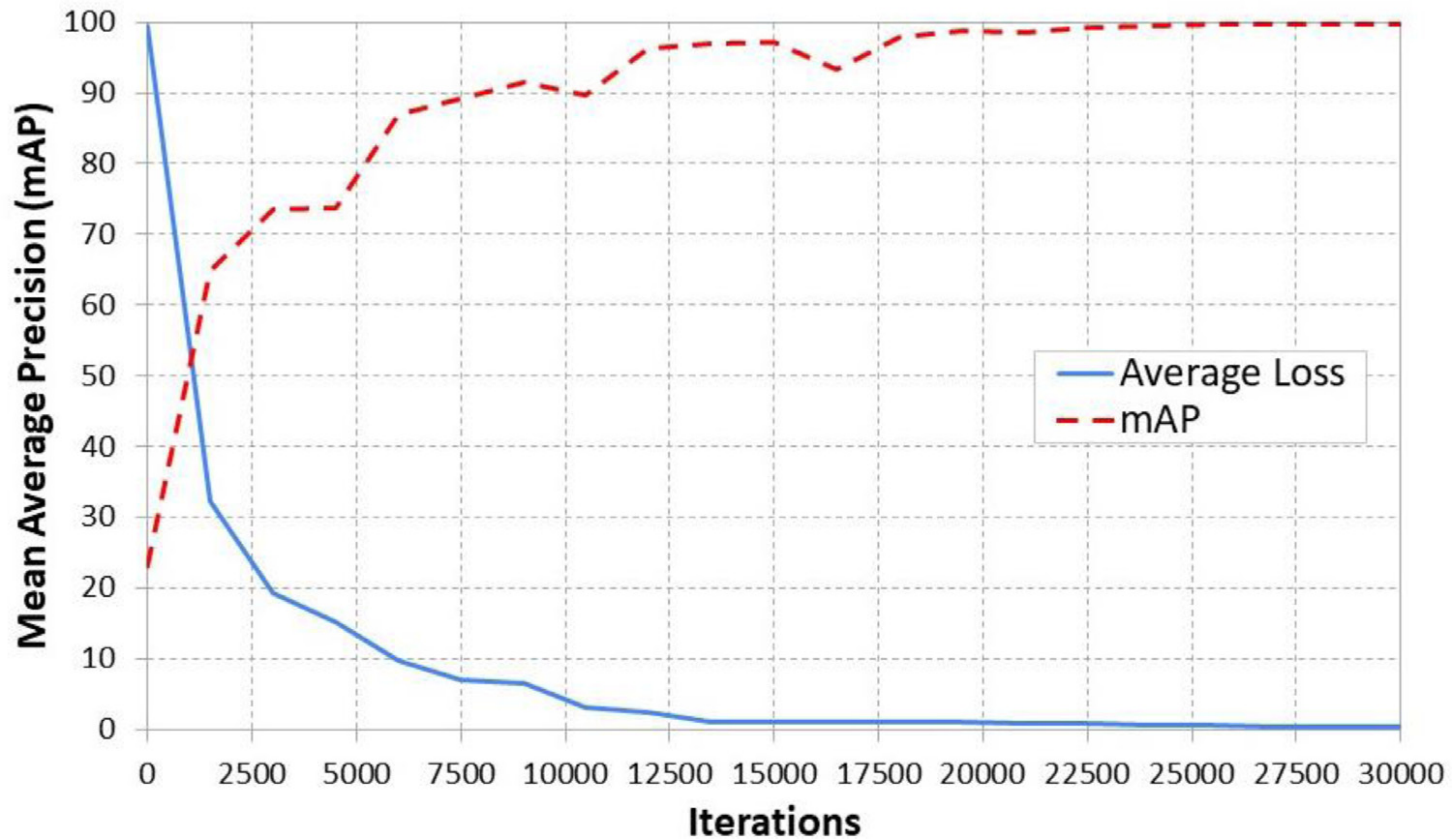
Mean-average precision chart of the YOLOv4 model [9] on the detection dataset

On the other hand, Fig. 5 illustrates the mean average accuracy and loss curve for the YOLOV4 detection model [9], which detects sign languages from images with a mean average precision of 99.9%. It is a highly accurate and generalized detection model.

The data must be annotated before training the YOLOv4 model. Each 'img-name.png' image is accompanied by an "img-name.txt" file that contains annotation information. The text file includes the image's class number, x-min coordinates, x-max coordinates, y-min coordinates, and y-max coordinates. Python and a couple of packages, such as lxml and pyqt5, are required to use this utility. After installing these packages, we launch the application and load images from the specified folder. Then, a bounding box is manually drawn around the object to be detected. The annotation format must be YOLO in order to be able to annotate the images. The bounding box is a precisely drawn rectangle surrounding the detection zone that is subsequently assigned a class. The coordinates of this box are determined after sketching the region. These coordinates must then be normalized depending on the image's height and width before being saved as the annotated document in the previously stated text format. The annotating procedure follows Algorithm 1.

**Algorithm 1:** 

*ImgList = getAllImginDir(path)* ***while****len(Img List)*≠*0 do* img = ImgList.pop() *class_no = getClassNo()* *x, y, width, height = gelCoordinates(Draw(img))* *x,y,width, height = this (x/width),(y/height),(width/img_width),(height/img_height)* *write(class_no, x, y, width, height) as txt* ***end while***

## Ethics Statements

In this dataset, all contributors voluntarily participated in its creation. Smartphone cameras captured all the hand-sign images. Using cameras, we only captured the hand gestures of the volunteers. In that case, there is no impact on our bodies. Therefore, there are no health-related ethical issues.

## CRediT authorship contribution statement

**Ayman Hasib:** Validation, Investigation, Data curation, Writing – original draft, Visualization. **Jannatul Ferdous Eva:** Validation, Investigation, Data curation, Writing – original draft, Visualization. **Saqib Sizan Khan:** Methodology, Validation, Investigation, Data curation, Writing – original draft, Visualization. **Mst. Nipa Khatun:** Data curation, Writing – original draft. **Ashraful Haque:** Data curation, Writing – original draft. **Nishat Shahrin:** Data curation, Writing – original draft. **Rashik Rahman:** Writing – review & editing, Supervision, Validation, Project administration. **Hasan Murad:** Supervision, Project administration. **Md. Rajibul Islam:** Writing – review & editing, Supervision, Project administration. **Molla Rashied Hussein:** Writing – review & editing, Supervision.

## Declaration of Competing Interest

The authors declare that they have no known competing financial interests or personal relationships that could have appeared to influence the work reported in this paper.

## Data Availability

BDSL 49: A Comprehensive Dataset of Bangla Sign Language (Original data) (Mendeley Data).
